# The Use of Polyhexanide and Betaine Combined Preparation in Adult Burn Care in Sri Lanka

**DOI:** 10.7759/cureus.67274

**Published:** 2024-08-20

**Authors:** Samitha Senevirathne, Gayan Ekanayake, Dishan Samarathunge, Oshan Basnayke

**Affiliations:** 1 Professorial Surgical Unit, National Hospital of Sri Lanka, Colombo, LKA; 2 Plastic and Reconstructive Surgery, National Hospital of Sri Lanka, Colombo, LKA

**Keywords:** mid-dermal burns, superficial partial-thickness burns, betaine, polyhexanide, burn injury

## Abstract

Biofilm formation over burn wounds has led to persistent wound infection, poor wound healing, and resistance to antimicrobial therapy. This process ultimately leads to prolonged hospital stays and increased cost of burn wound care, especially in developing countries. Hence, large-area biofilm-targeted therapy should be a mainstay in burn wound care. Polyhexanide is a polymer used as a disinfectant, and betaine is a surfactant. We report a patient managed with a combined preparation of the above two substances. A 44-year-old patient sustained a 22% superficial partial-thickness and mid-dermal burns on the back and right arm after a high-voltage electrocution injury. The patient was treated with dressings containing the above preparation and closely monitored for the healing stages of the burn wound. Complete wound epithelialization with healthy granulation tissue was achieved within 15 days. No surface wound swab culture became positive during the treatment. The patient did not develop any fever spikes, and the white blood cell count was maintained at less than 12,000 mm^-3^ with a C-reactive protein level below 50 mg/L. No surgical intervention was needed for further management of the wound. Polyhexanide and betaine combined preparation may be used effectively on the superficial partial-thickness and mid-dermal burns to prevent wound infection and improve granulation and epithelialization. However, high-quality comparative evidence is needed for the confirmation.

## Introduction

Biofilm is an aggregation of microcolonies that develop between a surface and liquid-based extracellular substance of microbial origin [1]. Biofilm formation in burn wounds has led to major treatment resistance, and 60% of burn-related mortality is attributed to biofilms [2]. Biofilms are associated with chronic inflammation, increased exudate, and increased resistance to antimicrobial therapy [3,4]. According to the manufacturer details, Prontosan (trade name; B. Braun Medical AG, Switzerland) is a combination of betaine, which is a surfactant, and polyhexamethylene biguanide, also known as polyhexanide, which is a preservative and adjuvant antimicrobial agent. It is available as a wound irrigation solution and in gel form. The wound irrigation solution consists of 0.1% polyhexanide and 0.1% betaine, and the gel form consists of 0.1% polyhexanide, 0.1% betaine, glycerol, and hydroxyethylcellulose. Although these preparations have been proven effective for chronic ulcers, the evidence for adult burn wound care is minimal [5,6]. In Sri Lanka, we see more accidental burns, which are household, and electrocution injuries are relatively rare. This report describes a patient with an electrocution burn injury who was managed with the above-mentioned wound irrigation solution and gel forms and their outcome. This case was reported to evaluate the effectiveness of Prontosan on adult burn wounds by studying the wound-healing process of a patient who had an electrocution burn injury managed with Prontosan. This particular brand was chosen as it is the only preparation available in the region where the study was carried out containing betaine and polyhexanide. Informed consent was obtained from the patient for case reporting and images.

## Case presentation

**Figure 1:**
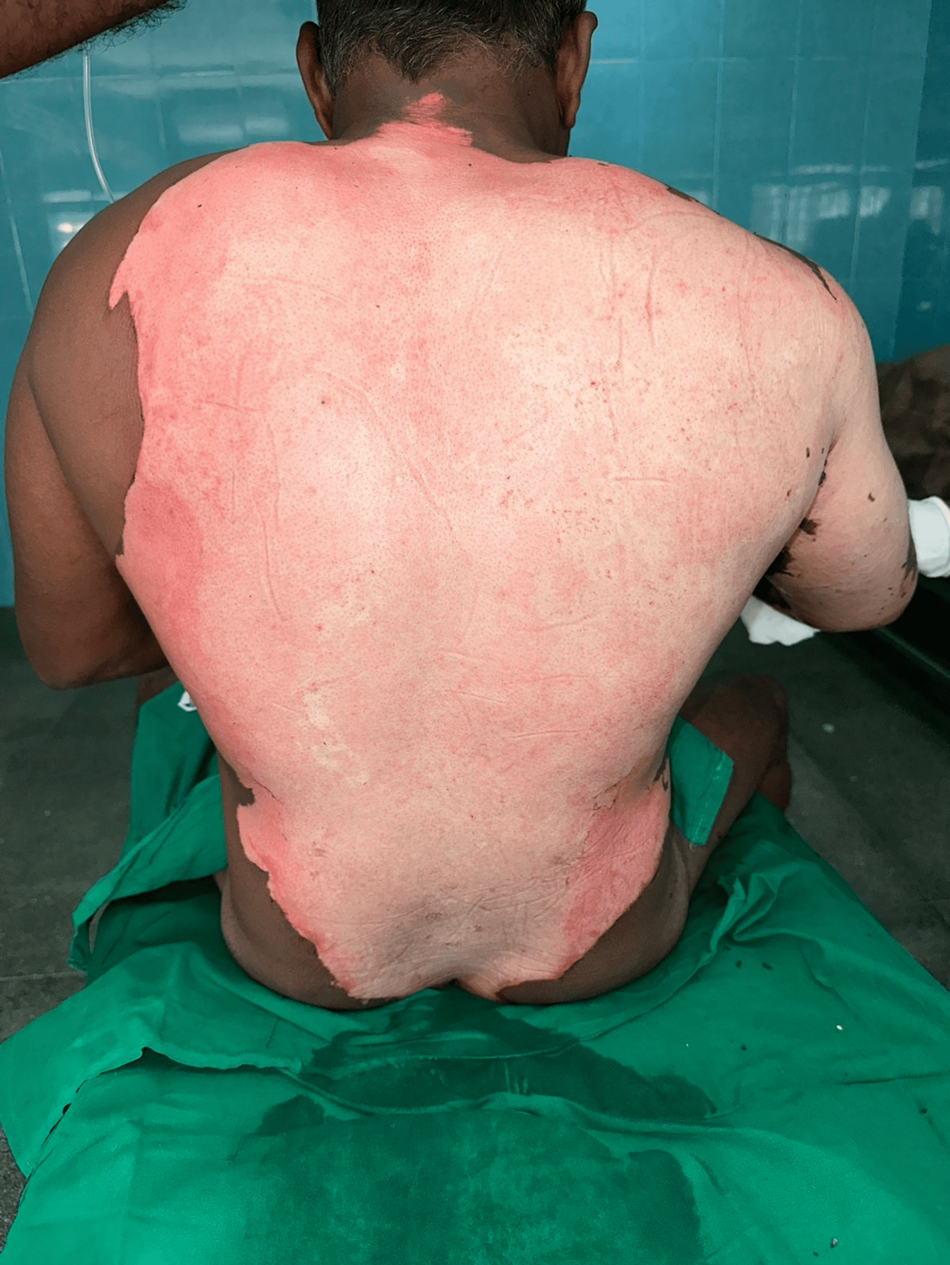
Initial appearance following burn scrub

**Figure 2:**
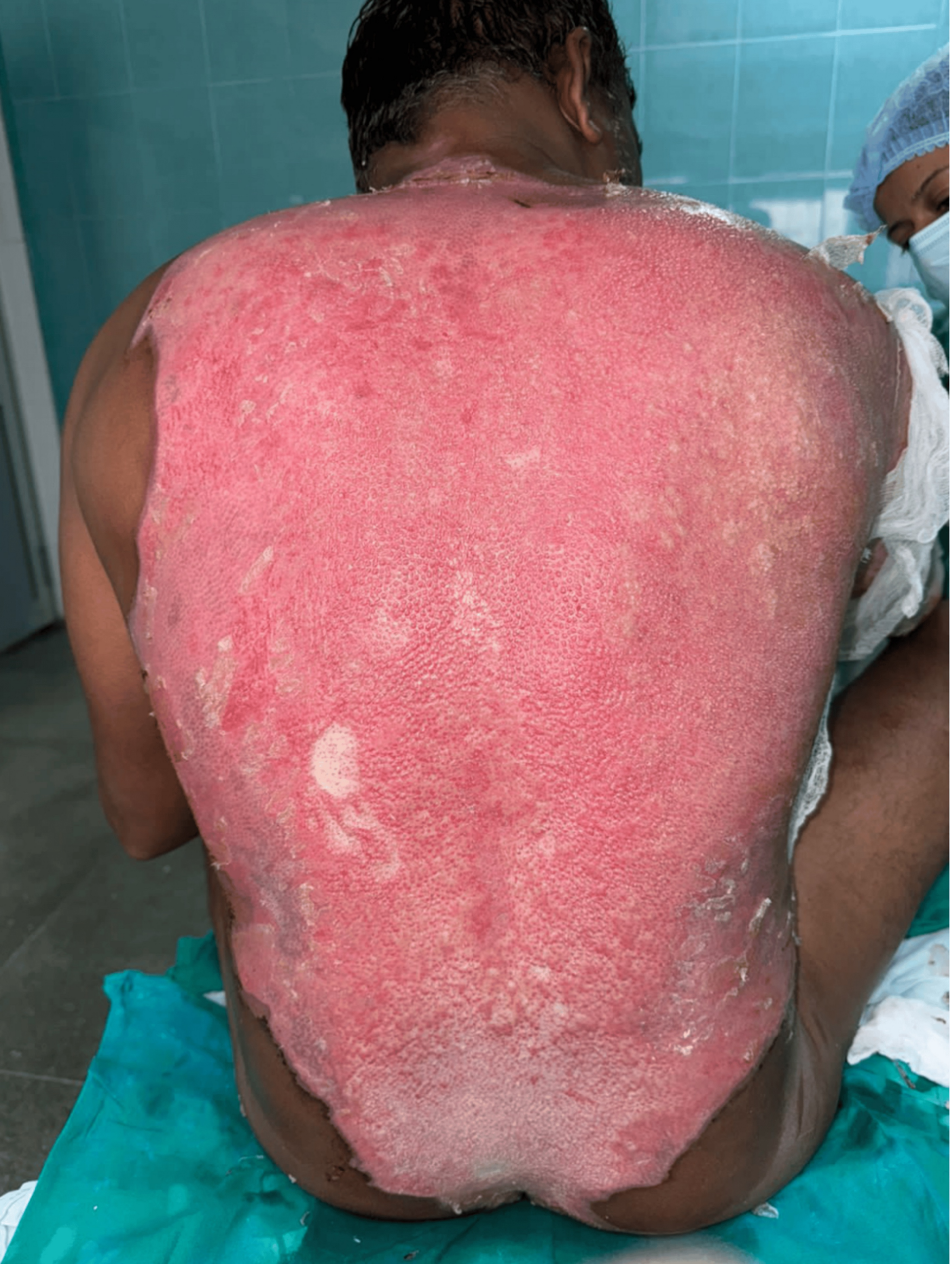
Day 1 after starting Prontosan

**Figure 3:**
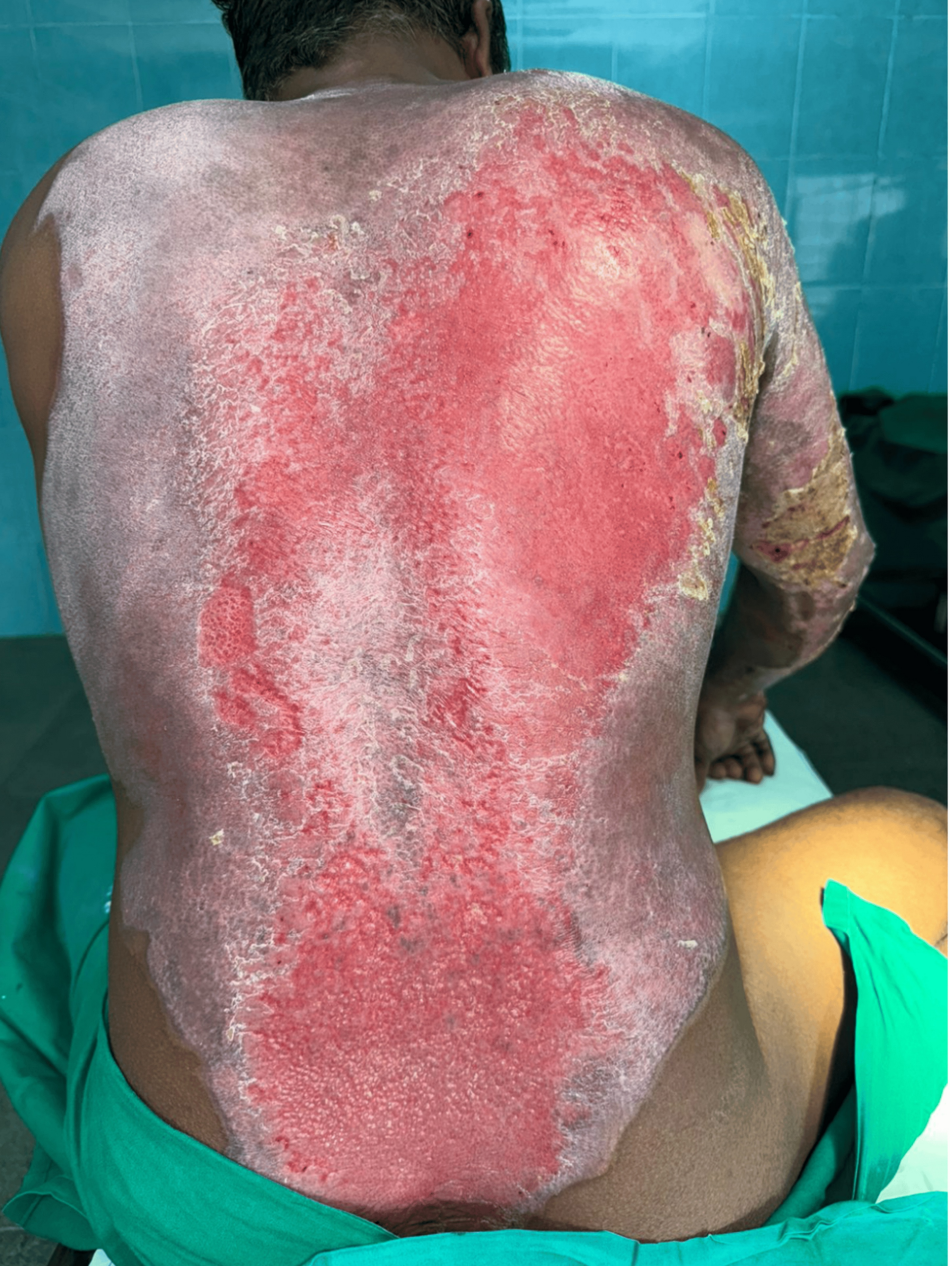
Day 7 after starting Prontosan

**Figure 4:**
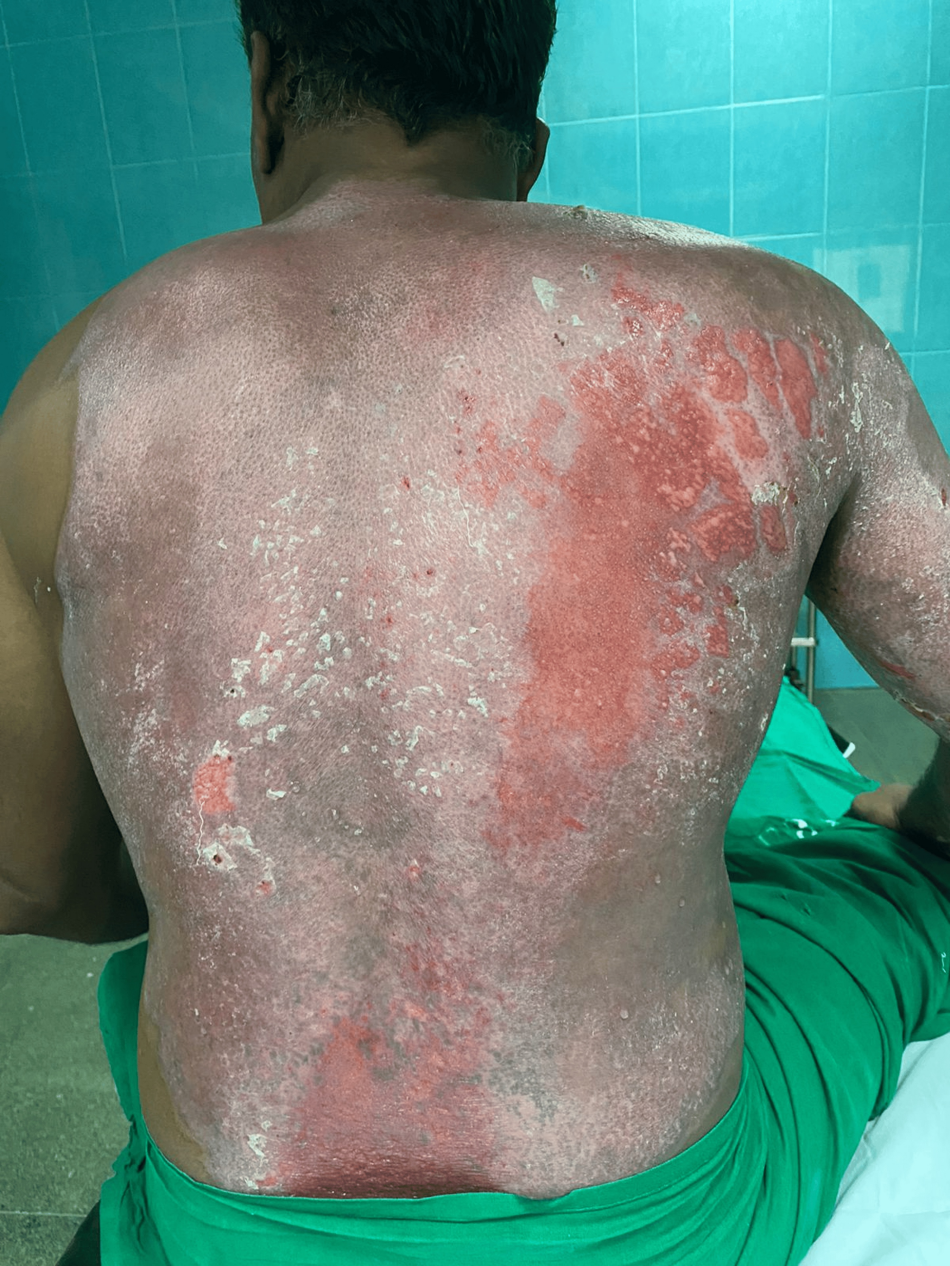
Day 11 after starting Prontosan

In the initial presentation, a 44-year-old American Society of Anesthesiologists 1 male patient who did not have other comorbidities presented to the accident and emergency service of the National Hospital of Sri Lanka in December 2023 following a high-voltage electrocution injury. Following initial management, according to the advanced trauma life support protocol, the patient was taken over to the burns unit and underwent a burn scrub under sedation with intravenous ketamine and fentanyl on the following day. He was found to have a mixed-depth burn involving superficial partial-thickness and mid-dermal burns over the back and right arm, with a total burn surface area of 22% (Figure 1). Initial burn dressing was done with 0.1% silver sulphadiazine, which is used as the routine dressing substance in Sri Lanka. During treatment, the patient had a similar dressing over the next five days once a day. As it was noted to have increased slough in the wound with Pseudomonas colonization, as confirmed by a surface wound swab culture sensitive to ceftazidime, it was decided to start the patient on Prontosan dressings. The adopted technique was a daily bath followed by Prontosan dressing daily, and the dressing frequency was reduced according to the healing process of the wound. During the dressing procedure, the wound was covered with Prontosan irrigation solution-soaked sterile gauze for five minutes and then rinsed. Later, a thin layer of Prontosan gel was applied over the wound and covered with the primary dressing. This dressing technique was in accordance with manufacturer recommendations. Wound dressings were done under analgesic cover of morphine, ketamine, and fentanyl. In the observation of the outcomes, it was noted that fine granulation with peripheral to central epithelialization appeared gradually, and the wound was completely healed in 15 days. During the treatment, there was no surface culture positivity, and white blood cell count was maintained at less than 12,000 mm^-3^ with a C-reactive protein (CRP) level of less than 50 mg/L. CRP values were 46, 44, and 32, respectively, done every fourth day. Clinically, the patient did not develop any fever spikes during the treatment. The patient was discharged at the end of January 2024. Figures 1-4 show the various stages of the wound-healing process. Upon discharge, the patient was started on burn scar treatment, including massaging techniques and compression garments. According to the unit protocol, the patient will be followed up at the outpatient burn scar management clinic for 18 months. The follow-up frequency will be two weeks, one month, three months, six months, 12 months, and 18 months after the discharge. On every visit, the patient is assessed by a burn injury specialized doctor, nursing officer, physiotherapist, and occupational therapist.

## Discussion

Burn wound care has become one of the most expensive categories among adult wound care. With a higher prevalence of severe burns, this poses a significant burden for health care. Therefore, it is vital to explore new knowledge of effective dressing material and their evidence basis. The use of Prontosan wound irrigation solution and gel in adult burn care is yet to be explored. A randomized controlled trial comparing the efficacy of Prontosan and silver sulphadiazine in treating partial-thickness burns has found that there is no difference in healing times, infection rates, bacterial colonization, and treatment cost between the two groups except the pain, which was less with Prontosan [7]. However, it is noted that when it comes to the South Asian region, the cost of burn care with Prontosan is higher than that of silver sulphadiazine. However, when the efficacy gap between Prontosan and silver sulphadiazine comes into the picture, the statement described above might have to be changed, considering the overall cost of care. A prospective, multicentered, noncomparative study assessing the efficacy of Prontosan gel on deep partial-thickness and full-thickness burns managed with split-thickness grafts has shown promising results in wound healing [8]. However, the same study has shown that Prontosan is not a substitute for split-thickness skin grafts. Therefore, the effect of Prontosan on the deep partial-thickness and full-thickness burns is questionable. Another study states that Prontosan is clinically and histologically superior in treating second-degree burns as it improves reepithelialization [9]. The study describes reepithelialization of second-degree burns managed only with Prontosan without plastic surgical intervention, which has been achieved within an average of 10 days, and compared to silver nitrate, no fibrin layer was noted on the wound. This might be due to biofilm disrupting the quality of Prontosan. A single-center randomized controlled study compared a biocellulose dressing containing polyhexanide with silver sulphadiazine in treating partial-thickness burns. The study found that polyhexanide necessitated less frequent dressing changes [10]. Burn dressing, though done under analgesic cover, is a traumatic experience for the patient, and frequent dressings might disrupt regenerating epithelium. Therefore, polyhexanide-containing gel forms like Prontosan are beneficial in terms of minimal disturbance to the patient compared to silver sulphadiazine, which usually needs daily dressing changes. A systematic and narrative study on topical agents on the pH of wounds describes that polyhexanide-containing preparations reduce the bacterial burden of wounds by reducing the surface pH [11]. A case series done on Prontosan gel form on chronic wounds states that Prontosan initiates wound bed preparation within two days of initiation of treatment, minimizing the slough and malodor with a 55% reduction in dressing frequency [12]. In summary, considering the above studies, Prontosan may positively impact adult burn wound care by increasing epithelial regeneration rate, reducing bacterial burden, minimizing pain and dressing frequency, aiding wound bed preparation, and reducing the overall cost of burn care. However, good quality evidence of the safety and efficacy of Prontosan in adult burn care in Sri Lanka and the south Indian region is almost difficult to find.

## Conclusions

This case report describes a patient who had a high-voltage electrocution mixed-depth burn of 22% total burn surface area involving the back and the right arm who had a satisfactory outcome in terms of avoiding the possible need of split thickness skin graft after managed with Prontosan wound irrigation solution and gel. We believe that Prontosan may be useful in managing mid dermal burns, which might get converted to chronic wounds. However further comparative high quality evidence is needed in order to improve patient outcomes with similar presentations.
